# One more rep! The case for resistance training in young cancer survivors

**DOI:** 10.3389/fonc.2023.1284052

**Published:** 2023-12-04

**Authors:** Nemanja Lakicevic, Salvatore Ficarra, Sonia Ortega-Gómez, David Jiménez-Pavón, Zoi Asimakopoulou, Apostolos Vantarakis, Paula Tavares, Vasco Vaz, Joshua Thaller, Sofia Papakonstantinou, Musa Kirkar, Francesca Glorioso, Antonio Palma, Antonino Bianco

**Affiliations:** ^1^ Sport and Exercise Sciences Research Unit, University of Palermo, Palermo, Italy; ^2^ MOVE-IT Research Group, Department of Physical Education, Faculty of Education Sciences, University of Cadiz, Cadiz, Spain; ^3^ Biomedical Research and Innovation Institute of Cadiz (INiBICA), Cadiz, Spain; ^4^ CIBER of Frailty and Healthy Aging (CIBERFES), Madrid, Spain; ^5^ Department of Public Health, Medical School, University of Patras, Patras, Greece; ^6^ Faculty of Sport Sciences and Physical Education and ICBR Faculty of Medicine, University of Coimbra, Coimbra, Portugal; ^7^ Department of Health Consulting, Research and Science, Outdoor Against Cancer, Munich, Germany; ^8^ Creative Thinking Development (CRE.THI.DEV), Rafina, Greece; ^9^ Centro Internazionale per la Promozione dell’educazione e lo Sviluppo (CEIPES), Palermo, Italy; ^10^ Lega Italiana per la lotta Contro i Tumori (LILT Palermo), Palermo, Italy

**Keywords:** cancer, resistance training, strength training, exercise oncology, muscle tissue

## Abstract

Resistance training is now seen as a powerful tool to improve the health and functionality of cancer survivors. Literature shows that it can be implemented both during and after cancer treatment, with the intent of preserving muscle mass in the former and increasing muscle mass in the latter case. However, currently available data on this matter are predominantly derived from adult cancer survivors (ACS), and it is questionable whether the exact same raining regimen should be implemented in young cancer survivors (YCS) given the unique challenges they experience throughout their disease trajectory. Therefore, the goal of this work is to distill the existing evidence on resistance training (RT) interventions in ACS and facilitate discussion on whether the same patterns of RT can be applied in YCS.

## 1 Introduction

Over the last several decades, evidence has shown us that resistance training (RT) is not just about feeling bulky and looking good but that there is more beyond aesthetics. The main target of RT, skeletal muscle, is now seen as an essential element of the complex network between hormonal, metabolic, and inflammatory pathways in addition to its innate function in the neuromuscular system (1, 2). Indeed, it is unsurprising then that the health benefits of RT are numerous and include reduced all-cause mortality and cardiovascular disease incidence, improved quality of life, improved mental health and physical functioning, and prevention of sarcopenia (3–8). In addition, the risks associated with regular RT are negligible compared to the benefits associated with this type of training (3). Likewise, novel studies indicate many benefits of RT in adult cancer survivors (ACS) (9), but it seems that ACS are rather sedentary both before (10) and after cancer treatment (11, 12). It is estimated that more than 70% of ACS are obese or overweight (13). Nevertheless, the most recent findings suggest that ACS can improve physical fitness through regular exercise (including RT) similar to adults unaffected by cancer (14). In addition, a recent meta-analysis showed that RT increases muscle strength independent of the cancer treatment type (15).

Despite exercise therapy, including RT, being appraised as the most effective adjunct therapy to prevent and treat a spectrum of late effects of cancer and therapy in young cancer survivors (YCS), there is stunningly poor evidence supporting the benefits of exercise based on YCS-focused research (16). Young cancer survivors are those who develop cancer between the ages of 15 and 39 and are recognized as a distinct population in the oncology community given the unique challenges they experience throughout their disease trajectory (17).

However, despite the accumulating data on RT in ACS, there is an ongoing debate about whether the same RT guidelines should be followed in the YCS RT regimen. Thus, the goal of this manuscript is to distill the existing evidence on RT interventions in ACS, describe employed patterns of RT used in the intervention studies, outline key benefits and potential pitfalls, and facilitate discussion on whether the same RT patterns can be applied in YCS.

## 2 RT design

As safety always comes first, it is important to outline that there is a consensus that under initial supervision and proper guidance, RT is safe in ACS (14). This has been shown in a plethora of studies, but it is very important to take into account whether RT is conducted during or after cancer therapy, as treatment-associated fatigue can be a limiting factor when performing RT, especially during treatment (18). Likewise, age, severity (stage) of cancer, and previous experience in RT need to be taken into account when considering RT design (19). Ideally, RT would be tailored to fit the needs of every patient with cancer, but in reality, this is often not the case, as many cannot afford personal trainers and are usually part of larger groups that follow identical protocols regardless of the previously mentioned factors. However, the social component of group training can be a potent tool in helping patients with cancer adhere to the RT protocol that was initiated during the intervention (20). In contrast, in case ACS or YCS want to perform training at home or any other setting of their choice, there are many zero-cost phone apps that offer audio-visual guidance on how to perform RT.

Despite growing data on RT in ACS, some authors claim that current approaches to RT prescription in this population can be seen as “basic and potentially underdeveloped” (21). Indeed, many randomized controlled trials (RCTs) dealing with cancer survivors still fail to report essential aspects of RT such as modes of progression, duration of RT sessions, and baseline fitness levels of subjects (21, 22). Thereby, caution should be taken when a certain study reports that RT was ineffective in a specific domain of interest, as this might be due to inadequate RT design rather than lack of efficacy of the RT itself (21).

However, certain common traits can be detected among higher-quality randomized control trials that have conducted RT in ACS (13, 14, 21). When designing RT for this population, it is advised to involve major muscle groups via six to eight exercises in a single session (on non-consecutive days). These exercises should be performed in one to three sets, 8–12 repetitions per set. To accustom novel exercisers, RT can be initially performed on machines and later by free weights, as the latter stimulates muscle tissue to a greater degree than machine-based exercises (23, 24). In addition, resistance bands or bodyweight exercises may be considered when a lack of resources or deconditioning may preclude exercise participation. Intensity should be matched to 60%–70% of 1 repetition maximum (1RM), whereby RT would be challenging but not exhausting. Progression can be carried out by increasing the weight or resistance in a given exercise, increasing the number of repetitions per set or the number of sets in total, reducing the break between the sets, or all of the above. However, progression should also be adjusted according to the subjective perception of RT; hence, tools like the Borg scale can be very useful to ensure that RT is challenging but not overwhelming.

### 2.1 Current recommendations for young cancer survivors

As RT studies on YCS are rather scarce, it seems widely accepted that this group should follow the guidelines from RT studies performed in ACS. In the following paragraphs, we will outline key components of RT implemented in ACS in the existing literature.

Using the appropriate individualization, RT may be used to target specific cancer and therapy symptoms. The current American College of Sports Medicine (ACSM) guidelines affirm that RT alone may be a valuable strategy to improve the health of ACS, although suggesting in the first place a combined moderate-intensity aerobic protocol (3 times/week for a minimum of 30 minutes) with the inclusion of 2 RT days (60% of 1RM, 2–3 sets of 8–15 repetitions per muscle group) (25). The Exercise & Sports Science Australia (ESSA) guidelines, supporting the benefits of exercise for ACS, emphasize the need to individualize multimodal moderate- to high-intensity exercise interventions (which is consistent with ACSM guidelines’ final considerations) (26).

In addition, the American Society of Clinical Oncology (ASCO) guidelines for ACS undergoing treatments indicate aerobic and RT as a strategy to dampen treatment side effects (27). Interestingly, these guidelines (including diet and weight management indications) highlighted the paucity of evidence supporting weight loss interventions (or weight gain avoidance) to improve patients’ health (27). Therefore, the anabolic potential of RT could be, however, supported to avoid cachexia, which is diagnosed in approximately 50% of patients with cancer (15), and deconditioning. This indication may also be extremely relevant for older patients who may have faced muscle mass loss before diagnosis and therapies and present higher cachexia levels than YCS (28, 29).

Until more data on studies specifically designed for YCS are available, it is reasonable to follow RT guidelines for ACS while being cautious of unique needs and barriers that this population might face. Engaging in RT and other forms of exercise is particularly important for YCS given their higher 5-year survival rates (82.5%) and the greater potential for years of productive life lost per individual than people diagnosed after the fourth decade (16).

## 3 Future perspectives

RT and its anabolic effect may be a strategy not only to avoid muscle mass loss (particularly in the older cancer population) but also to maintain higher adherence, which should be promoted through individualization. Tailoring the intervention is extremely relevant when considering the difference between those who are undergoing therapy and those who completed treatments with curative intent. In the first population, RT strategies should be implemented to avoid excessive muscle mass loss and deconditioning, aiming to maintain patients’ physical function, rather than improving it. For those who completed all treatments, RT could be essential not only to recover from therapeutic side effects but, once recovery is completed, also to improve physical fitness levels, similar to what is conducted for healthy individuals.

Although individualization could be the key to exercise prescription on cancer populations, it is also worth noting that certain types of cancer have been understudied in the exercise oncology field and that guidelines commonly refer to randomized controlled trials on commonly and less detrimental cancer types (e.g., breast, colorectal, and prostate cancers). Exercise specialists should tailor RT intervention for patients with cancer after oncologist approval and know the risks that exercise may cause along with the safety procedures to avoid those risks. As the exercise was recently adopted as a standard of cancer care in Australia and will likely be adopted in the USA and Canada (30), thorough guidelines on implementing RT in YCS are imperative.

## 4 Discussion

Although the literature on exercise oncology has grown immensely in recent years, there are certain issues in this field that need to be addressed. As stated previously, precise reporting on the details of the RT interventions is lacking in many instances. To provide valid and reliable RCTs that can be replicated in various settings, methodological quality, i.e., detailed study design description, should be mandatory and requested by the reviewers and editors of journals. Vague descriptions of RT patterns employed in RCTs can lead to inaccurate conclusions and inappropriate delivery in clinical and community settings. To prevent this, scientists have developed a standardized method for reporting exercise programs that require a thorough description of essential aspects of exercise interventions (31). This tool can markedly improve the ability to accurately analyze and replicate RT interventions.

In keeping with this theme, the heterogeneity of the existing RCTs needs to be discussed. Indeed, studies lasting 12–52 weeks of an intensity of 40%–80% 1RM and different progression modes (if reported) are likely to yield vastly different results in the outcomes measured (15). In addition, adherence to RT protocols is either poorly reported or not reported at all in currently available studies (32). Likewise, there are also major issues with the standardization of procedures for body composition assessment in ACS, making it unclear to delineate the exact effects of RT on body composition as opposed to errors in methodology or assumption (32). Moreover, we accounted that available literature on RT and its effects on muscle mass in ACS rarely if ever included reporting on dietary patterns of cancer survivors despite muscle mass commonly being the most important outcome observed. Data on muscle wasting and diet are still in their infancy, and certainly, more studies need to be conducted in this field. Finally, data on the follow-up after RT interventions are rather scarce, making it unclear whether cancer survivors adhere to their RT regimen after the intervention.

Future studies should investigate the hypertrophic potential of young cancer survivors (33). Based on the data available from the healthy population, age might be a limiting factor when it comes to muscle gains as a result of RT (34). Thus, it would be interesting to see the differences in RT-caused muscle gains in young versus older cancer survivors under standardized conditions. Furthermore, researchers should consider reporting male and female data separately, as men commonly have a significantly greater absolute increase in muscle volume compared to women following RT (35).

On a broader scale, medical staff might be a key figure in RT adherence, as patients reported a higher amount of physical activity (PA) at 2–3 years post-diagnosis in patients with cancer who recalled receiving physical activity advice from a health care professional after diagnosis compared to those who did not recall receiving the same advice (36). Indeed, data show that only 51% of health professionals reported giving PA advice to their patients, while 36% declared to be unaware of any lifestyle guidelines for cancer survivors, and approximately half (49%) were aware of PA guidelines (37).

We acknowledge that other forms of exercise induce numerous health benefits in ACS and YCS, but the focal point of this manuscript was RT and muscle tissue, which seems to be the tissue most aggravated by cancer and cancer treatments. To our knowledge, RT is by far the most effective way to preserve and increase muscle mass in ACS, and thus RCTs on the effects of RT on YCS are urgently needed.

## Data availability statement

Publicly available datasets were analyzed in this study. This data can be found here: Data will be provided upon reasonable request.

## Author contributions

NL: Conceptualization, Data curation, Writing – original draft, Writing – review & editing. SF: Conceptualization, Data curation, Investigation, Writing – original draft, Writing – review & editing. SO: Conceptualization, Supervision, Writing – review & editing. DJ: Conceptualization, Investigation, Writing – review & editing. ZA: Methodology, Supervision, Writing – review & editing. AV: Methodology, Supervision, Writing – review & editing. PT: Funding acquisition, Project administration, Writing – review & editing. VV: Funding acquisition, Project administration, Writing – review & editing. JT: Funding acquisition, Resources, Writing – review & editing. SP: Funding acquisition, Resources, Writing – review & editing. MK: Formal Analysis, Project administration, Writing – review & editing. FG: Supervision, Validation, Writing – review & editing. AP: Conceptualization, Writing – original draft, Writing – review & editing. AB: Data curation, Investigation, Writing – original draft, Writing – review & editing.
